# Identification of genes which regulate stroma-dependent *in vitro* hematopoiesis

**DOI:** 10.1371/journal.pone.0205583

**Published:** 2018-10-11

**Authors:** Pravin Periasamy, Vinson Tran, Helen C. O’Neill

**Affiliations:** 1 Department of Microbiology and Immunology, Yong Loo Lin School of Medicine, National University of Singapore, Singapore; 2 Division of Biomedical Science, Research School of Biology, The Australian National University, Canberra, Australia; 3 Clem Jones Centre for Regenerative Medicine, Faculty of Health Sciences and Medicine, Bond University, Gold Coast, Queensland, Australia; Emory University, UNITED STATES

## Abstract

Cultured splenic stroma has been shown to support *in vitro* hematopoiesis in overlaid bone marrow and spleen progenitors. These co-cultures support longterm production of a novel dendritic-like cell type along with transient production of myeloid cells. They also maintain a progenitor cell population. The splenic stromal lines 5G3 and 3B5 have been identified as a supporter and a non-supporter of hematopoiesis. Based on their gene expression profile, both 5G3 and 3B5 express genes related to hematopoiesis, while 5G3 cells express several unique genes, and show upregulation of some genes over 3B5. Based on gene expression studies, specific inhibitors were tested for capacity to inhibit hematopoiesis in co-cultures. Addition of specific antibodies and small molecule inhibitors identified VCAM1, CXCL12, CSF1 and SPP1 as potential regulators of hematopoiesis, although both are expressed by 5G3 and 3B5. Through inhibition of function, SVEP1 and ALDH1 are also shown here to be deterministic of 5G3 hematopoietic support capacity, since these are uniquely expressed by 5G3 and not 3B5. The achievement of inhibition is notable given the dynamic, longterm nature of co-cultures which involve only small numbers of cells. The alternate plan, to add recombinant soluble factors produced by 5G3 back into 3B5 co-cultures in order to recover *in vitro* hematopoiesis, proved ineffective. Out of 6 different factors added to 3B5, only IGF2 showed any effect on cell production. The identification of differentially expressed or upregulated genes in 5G3 has provided an insight into potential pathways involved in *in vitro* hematopoiesis leading to production of dendritic-like cells.

## Introduction

Multiple dendritic cell (DC) subsets are present in spleen under steady-state and inflammatory conditions. DC precursors continually seed spleen from bone marrow where they develop into the well characterised cDC and pDC subsets [1]. Here we investigate splenic stromal lines which support *in vitro* hematopoiesis to produce novel dendritic-like cells following co-culture with hematopoietic progenitors from bone marrow or spleen [2–4]. The main subset of dendritic-like cells produced have been characterised for their distinct phenotype and functional capacity [5–7], and *in vivo* equivalent subsets have been identified in both mouse [8, 9] and human [10]. Mutant mouse studies have identified their progenitor origin as spleen rather than bone marrow. This novel subset is still produced in *c-Myb*^*E308G*^ mutant mice where development of bone marrow-derived dendritic and myeloid cells is compromised [11].

The importance of splenic stromal cells in hematopoiesis was first demonstrated for spleen-derived long-term cultures (LTC) which continually support myelopoiesis for years [12, 13]. The spleen stromal cell microenvironment maintains progenitor cells and supports restricted differentiation [14, 15]. Subsequent studies involved the heterogeneous spleen stromal cell line STX3 [12, 16] derived from one LTC that had ceased production of hematopoietic cells. Gene profiling of STX3 compared with the 2RL22 lymph node stroma, led to description of STX3 as an immature mesenchymal cell type which did not express mature endothelial cell markers but weakly formed tube-like structures in Matrigel [16, 17]. The STX3 stromal cell line was cloned to give multiple cell lines [18] which were each characterised in terms of morphology and ability to support DC hematopoiesis *in vitro*. This study identified the cloned stromal line 5G3 as a supporter of hematopoiesis, and 3B5 as a non-supporter. 5G3 has been characterised in detail in terms of its hematopoietic support capacity [2, 6, 19, 20].

Here, the 5G3 supporter and the 3B5 non-supporter lines have been compared through transcriptome analysis to identify of differences in gene expression to determine genes that may be critical determinants of *in vitro* hematopoiesis. Identification of differentially expressed or upregulated genes is a powerful approach for detecting novel genes and novel molecular pathways indicative of specific functional potential. Several genes have been identified which encode potential hematopoietic regulators. Their importance in hematopoiesis has been tested through application of available inhibitors to co-cultures to determine importance for hematopoietic output.

## Materials and methods

### Animals

Specific pathogen-free C57BL/6J (*H-2K*^*b*^) mice aged 6 weeks were obtained from the John Curtin School of Medical Research (JCSMR: Canberra, ACT, Australia). Mice were housed and handled according to protocols approved by the Animal Experimentation Ethics Committee at the Australian National University (ANU: Canberra, ACT, Australia).

### Cell culture

The continuous stromal cell lines 5G3 and 3B5 have been previously described [21, 22]. They were cloned from an original STX3 splenic stroma [13, 23, 24], established from a longterm culture (LTC) of B10.A(2R) mouse spleen which had ceased production of DC over time due to loss of hematopoietic cells. Stroma was maintained by scraping cells for passage into a new flask and culture at 37°C in 5% CO_2_ in air in Dulbecco’s modified Eagle’s medium (DMEM) supplemented with 10% fetal calf serum, 5 x 10^-4^M 2-mercaptoethanol, 10mM HEPES, 100U/ml penicillin, 100ug/ml streptomycin, 4mg/L glucose, 6mg/L folic acid, 36mg/L L-asparagine, 116mg/L L-asparagine hydrochloric acid (sDMEM). These were frozen down within 4–5 passages of cloning, and stromal lines passaged only 4–5 times after thawing for use in experiments.

### Isolation of bone marrow progenitors

Mice were killed by cervical dislocation. Bone marrow was obtained from femurs and tibias by flushing cells from the bone cavity using a syringe filled with sDMEM. Bone marrow suspensions were dissociated by forcing tissue through a fine wire sieve. In preparation for antibody-mediated magnetic cell depletion, cells were washed twice with 10ml labelling buffer [degassed PBS pH7.2, 0.5% bovine serum albumin and 2mM EDTA] by centrifugation at 300g and 4°C for 10 minutes. A cell pellet was resuspended in 40μl of labelling buffer containing a cocktail of biotin-labelled antibodies specific for hematopoietic lineage markers CD5, CD45R, CD11b, Gr-1 (Ly-6G/C), 7–4, and Ter-119 (10μl per 10^7^ cells) (Lineage depletion kit, Miltenyi Biotec: North Ryde, NSW, Australia) to which was added antibody to the DC marker CD11c (BioLegend: San Diego, CA, USA), for 10 minutes on ice. Following this, 30μl of labelling buffer and 20μl of MACS^®^ anti-biotin microbeads (Miltenyi Biotec) were added to cells and incubated on ice for a further 15 minutes. Cells were washed with 2ml of labelling buffer followed by centrifugation at 300g and 4°C for 10 minutes. Cells were resuspended in 500μl of labelling buffer and transferred to a MACS^®^ MS column (Miltenyi Biotec) and placed in a SuperMACS^®^ II Separator (Miltenyi Biotec). Cells which bind biotinylated antibody and the MACS^®^ anti-biotin microbeads are retained in the column. The MACS^®^ MS column was then washed three times with 500μl of labelling buffer and flow through cells collected. Cells were washed twice with sDMEM by centrifugation at 300g and 4°C for 5 minutes. An aliquot of the lineage depleted (Lin^-^) cell population was tested for the presence of lineage positive cells by antibody staining and flow cytometric analysis.

### Stromal cell co-cultures

The capacity of stromal cells to support hematopoiesis was assessed by overlay of Lin^-^ bone marrow cells above stromal cell monolayers grown to 80–90% confluency followed by co-culture for several weeks. Lin^-^ bone marrow cells were plated at 1–5 x 10^4^ cells/ml above the stromal monolayer in 25cm^2^ flasks. Co-cultures were held at 37°C, 5% CO_2_ in air and 97% humidity. At 7-day intervals, non-adherent cells were collected by gently shaking the flask with removal and replacement of supernatant. Cell yield was determined by counting using Trypan blue exclusion staining and cells analysed for surface marker expression by antibody staining and flow cytometric analysis. Specific inhibitors or growth factors were added into some co-cultures using a concentration range recommended by the supplier. These are listed in Tables 1 and 2. Additives were replenished at biweekly medium change.

**Table 1:** Recombinant factors added to 3B5 co-cultures.

Recombinant factor (Supplier)	Factor specificity
SFRP2 (R&D Systems)	Homologue of Frizzled; acts as a modulator of WNT signalling
RSPO2 (R&D Systems)	Activator for WNT/β-catenin signalling
IGF2 (R&D Systems)	Growth factor that binds to IGF1
POSTN (R&D Systems)	An adhesion molecule and tumour suppressor
WNT5a (R&D Systems)	Member of the non-canonical WNT pathway
CCL8a (Biolegend)	Chemokine that acts as an antagonist of CCR8

*Biolegend (San Diego, CA, USA); R&D Systems (Minneapolis, MN, USA).

**Table 2:** Inhibitors tested in 5G3 co-cultures.

Inhibitor (Supplier)*	Inhibitor Activity
DEAB (Stem Cell Technologies)	ALDH1 expressed by 5G3 and HSPC
GW2580 (Biovision Incorporated)	CSFR1, receptor for CSF1 produced by stroma
Anti-CXCR7 Mab (Biolegend)	CXCR7 on HSPC, receptor for CXCL12 produced by stroma
Anti- ITGA9 serum (LifeSpan BioSciences)	ITGA9 on HSPC, ligand for SVEP1 expressed by 5G3, and SPP1/TNC expressed by stroma
Anti-CD11a Mab (Biolegend)	CD11a on HSPC, ligand for CLCA1 expressed by 5G3
Anti-CD44 Mab (Biolegend)	CD44 on HSPC, receptor for SPP1/TNC expressed by 5G3
Anti-CD49d Mab (Biolegend)	CD49d on HSPC, ligand for VCAM1/FN expressed by stroma

*Biolegend (San Diego, CA, USA); Biovision Incorporated (Milipitas, CA, USA); Lifespan Biosciences (Seattle, WA, USA); Stem Cell Technologies (Vancouver, BC, CA).

### Microscopy

The morphology of stromal cells was monitored microscopically before and after addition of overlay cells. Cultures of stroma alone, or cocultures of Lin^-^ bone marrow cells above stroma were photographed using a DM IRE2 inverted research microscope (Leica: North Ryde, NSW, Australia) equipped with DFC digital camera (Leica) to obtain phase contrast photomicrographs. Images were processed using Leica IM software v4.0.

### Flow cytometry

For analysis of cell surface marker expression and subsequent subset delineation, cells were stained with antibodies diluted in fluorescence-activated cell sorting (FACS) buffer (DMEM/0.1% sodium azide/1% fetal calf serum). Antibody specific for FcγII/IIIR (CD32/CD16) (eBiosciences: San Diego, CA, USA) was used at 5 *μ*g/10^6^ cells in 1ml to block non-specific antibody binding. Fluorochrome or biotin-conjugated antibodies for CD11b (M1/70), CD11c (N418), MHC-II (AF6-120.1), CD45RB (C363.16A), Sca-1 (D7), c-Kit 2B8), streptavidin-PE-Cy7, streptavidin-PE, streptavidin-FITC and isotype control antibodies were purchased from either Biolegend or eBiosciences (San Diego, CA, USA). All antibodies were titrated to determine concentration giving minimal saturating binding. Cells labelled with fluorochrome-conjugated antibodies were analysed in both multicolour and single colour staining protocols. Discrimination of live cells utilized staining with 1μg/ml propidium iodide (PI) (Sigma-Aldrich: Castle Hill, NSW, Australia). Isotype matched control antibodies or fluorescence minus one (FMO) controls were used to set gates to delineate specific antibody binding. Flow cytometric analysis was carried out using BD FACSDiva (Becton Dickinson) and FlowJo software (Tree Star: Ashland, OR, USA).

Specific antibodies used in inhibition studies were titrated before use in co-cultures through binding to control Lin^-^ bone marrow cells, at concentrations of 0.3, 1 and 3 μg/mL. These are listed in Table 2. Median fluorescence intensity (MFI) was calculated as the net change in median channel fluorescence for specific antibody above isotype controls. Anti-CD11a, anti-CD44 and anti-CD49d antibodies bound strongly to target molecules on Lin^-^ bone marrow cells, giving shifts in median fluorescence intensity between 300 and 13000. Anti-CXCR7 and anti-ITGA9 bound to only small numbers of cells amongst Lin^-^ bone marrow cells and so were titrated on hematopoietic stem cells (HSC) sorted from Lin^-^ bone marrow as the Lin^-^ Sca-1^+^c-Kit^+^ subset.

### Transcriptome analysis

Total RNA was isolated from stromal cell lines, and concentration and purity determined spectrophotometrically. Double stranded cDNA was synthesized from RNA in a two-step process. The first strand of cDNA was synthesis using T7-(dT)_24_ primers and Superscript II reverse transcriptase (Invitrogen Life Technologies: Mount Waverley, VIC, Australia). This was followed by second strand cDNA synthesis. Double-stranded DNA was then purified using phenol-chloroform together with phase-lock gels (Brinkmann Instruments: Westbury, NY, USA). *In vitro* transcription and biotin labelling were performed using the BioArray High Yield RNA Transcript Labelling Kit (Affymetrix: Santa Clara, CA, USA). cRNA was purified on RNeasy Spin columns (Qiagen, SABiosciences: Valencia, CA, USA), fragmented, and labelled with biotin. Labelled cRNA was then hybridized to Murine Genome 430v2 genechips (Affymetrix) following the manufacturer’s instructions. They were washed followed by staining on the fluidics station (Affymetrix), ahead of scanning and image analysis using a Gene Array Scanner (Affymetrix). Scanned images of genechips were processed using Microarray Suite 5.0 software (MAS5.0; Affymetrix). Data files were prepared in Microsoft Excel containing probeset numbers, signal values and p-values. Further analysis involved extraction of data according to set criteria. The preparation of label, hybridisation to genechips, scanning, data compilation and basic analysis was performed by staff in the Biomolecular Resources Facility (JCSMR: Canberra, ACT, Australia).

### Real-time polymerase chain reaction

Total RNA was isolated from stromal cell lines using the RNeasy mini kit following the manufacturer’s protocol (Qiagen). RNA was purified using the genomic DNA elimination mix, and concentration and purity determined spectrophotometrically. Following this, Buffer BC3, Control P2, Reverse Transcriptase mix and RNase-free water were added in ratios of 4:1:2:3 for preparation of cDNA. Incubation proceeded for 15 mins at 42°C for denaturation, then for 5 mins at 95°C to convert RNA into cDNA. Subsequently, cDNA was diluted in RNase-free-water for use in Realtime PCR reactions. Equal volumes of cDNA and primer (10μM stock) were mixed. Primers were purchased from SABioscience (Frederick, MD 21703, USA). These included: *Abcg1* (PPM03895A); *Actb* (PPM02945A); *Aldh1* (PPM04398F); *Apod* (PPM05327F); *Ccl8* (PPM03165A); *Clca1* (PPM04051E); *Csf1* (PPM03116C); *Ctla2a* (PPM27745A); *Cxcl12* (PPM02965E); *Igf2* (PPM03655A); *KitL* (PPM02983C); *Ms4a4d* (PPM24757A); *Postn* (PPM34333E); *Rspo2* (PPM32746A); Serpinα3n (PPM05779F); *Sfrp2* (PPM03616C); *Spp1* (PPM03648C), *Svep1* (PPM05259A); *Wnt5a* (PPM04748C). The cDNA/primer mixture was then added to RT^2^ SYBR Green Mastermix and RNase-free water in a ratio of 1:6.25:5.25, respectively. Samples were loaded on to a LightCycler 480 (Roche: Castle Hill, NSW, Australia). The cycling conditions used were: 1 cycle for 10 mins at 95°C to activate DNA Taq Polymerase, followed by 45 cycles of 15 sec at 95°C for extension, and then 1 min at 60°C for fluorescence data collection.

Data analysis involved LightCycler 480 software v.1.2.9.11. (Roche). The Absolute Quantification (2^nd^ derivative max) method was used to obtain a cross point value (C_p_), referred to as the cycle threshold (C_t_). C_p_ is the point of maximal increase in fluorescence emitted by a single PCR reaction within the log-linear phase. To further analyse data, C_t_ values for genes of interest (GOI) along with housekeeping genes (HKG) were imported into Excel (Microsoft: Redmond, WA, USA). ΔC_t_ = C_t_ (GOI)–C_t_ (HKG) was calculated, and the average ΔC_t_ taken from quadruplicate experiments. To calculate the fold change between two samples, the calculation 2^-ΔCt^ (Sample 1) / 2^-ΔCt^ (Sample 2), was used. The resulting value corresponds to the relative difference in mRNA quantity between two samples. The presence of an amplified product was confirmed by gel electrophoresis.

### Statistical tests

When replicates were available, data are presented as mean ± standard error (SE) for sample size n. The Wilcoxon Rank Sum test has been routinely used to test significance (p≤0.05).

In experiments involving addition of inhibitors (or replacement factors) where very small numbers of progenitor cells were available, a titration of additives was performed in preference to replication of tests of additive at just one concentration. A significant inhibitory (or enhancement) effect was indicated by the outcome of even or decreasing (increasing) cell production over 4 dilutions of additive. The null hypothesis is that cell production is random across the titration, and the alternative hypothesis is that decreasing (or increasing) cell production occurs with increasing concentration of additive. The probability of an ordered reducing (or expanding) sequence for cell production over four different dilutions is 1/24 or 0.0417 which is significant at p≥0.05.

## Results

### Differential support of hematopoiesis by splenic stroma

**Fig 1:**
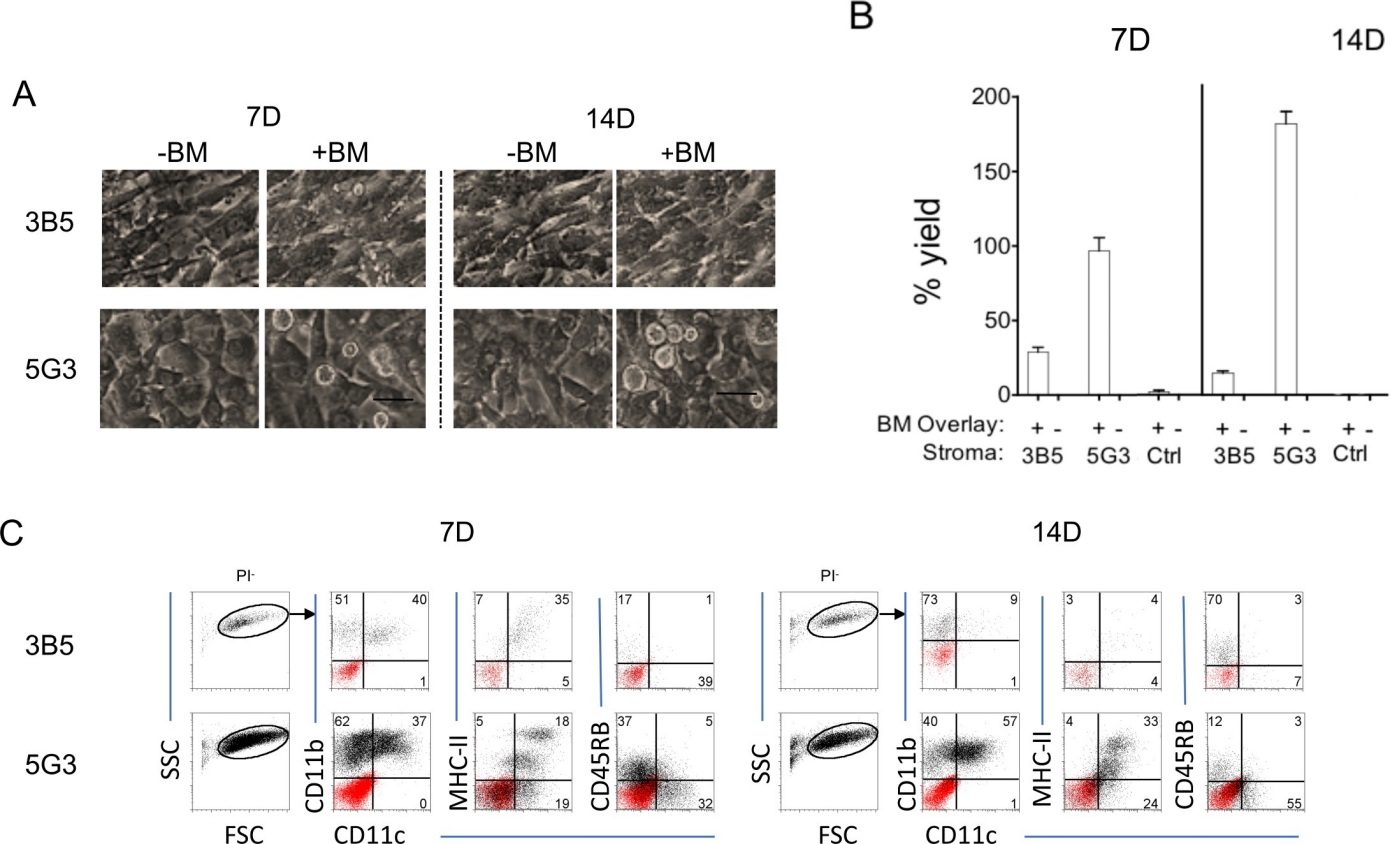
Comparison of splenic stromal lines for support of hematopoiesis. Co-cultures were established by overlay of Lin^-^ bone marrow cells on 3B5 and 5G3 stroma in 3 replicate experiments involving the same starting cell number. ‘Stroma only’ and ‘Lin^-^ bone marrow only’ cultures (not shown) served as controls. (A) Co-cultures were photographed at 7 and 14 days under phase contrast microscopy (Objective 20x, bar 100μm). (B) Non-adherent cells were collected after 7 and 14 days, and live cell recovery (% yield) estimated on the basis of input cell number. Data are presented as mean ± standard error (n = 3). Percent yield from 3B5 co-cultures is statistically different from 5G3 co-cultures, and control cultures of only Lin^-^ bone marrow at both 7 and 14 days (p≤0.05). (C) Non-adherent cells collected at 7 and 14 days were analysed flow cytometrically after gating live (PI^-^) cells. The phenotype of cells gated on forward and side scatter (FSC, SSC) was determined using antibody staining to detect expression of CD11c, CD11b, MHC-II and CD45RB. Isotype control antibodies were used to detect non-specific binding shown as a red overlay, and to set cross-hairs on bivariate antibody plots. Numbers in quadrants represent % positive cells.

Photomicrographs showed that 5G3 stroma displayed cobblestone morphology, while 3B5 stroma was more elongated and displayed a spindle-shaped fibroblastic morphology (Fig 1A). 3B5 and 5G3 have different capacity to support myelopoiesis in overlaid Lin^-^ bone marrow progenitors. Large non-adherent cells were detectable at 7 and 14 days above 5G3 but not above 3B5 (Fig 1A). Cocultures of only Lin^-^ bone marrow cells showed no viable cells after 14 days (not shown). Cell yield (%) determined by Trypan blue exclusion of recovered non-adherent cells at 7 and 14 days showed that 5G3 produced a significantly higher yield of live cells relative to input cell number than did 3B5 (Fig 1B). 3B5 co-cultures were only weakly able to support hematopoiesis *in vitro*, and less than 20% of input cells was recovered after 14 days. No live cells were recovered from ‘stroma only’ control cultures (Fig 1B).

Flow cytometry was used to distinguish live cells produced in co-cultures and to analyse their cell surface phenotype. Non-adherent cells produced in Lin^-^ bone marrow co-cultures were collected at 7 and 14 days and stained with antibodies for CD11c, CD11b, MHC-II and CD45RB. Ahead of flow cytometry, cells were stained with PI to identify live (PI^-^) cells. After gating to remove debris on a forward scatter (FSC) versus side scatter (SSC) plot, DC were identified as CD11b^+^CD11c^+^ and myeloid cells as CD11b^+^CD11c^-^. MHC-II and CD45RB were used to identify mature and any regulatory DC amongst CD11c^+^ cells, respectively (Fig 1D). CD11b^+^CD11c^+^MHC-II^+^ cells produced are phenotypically similar to CD8α^-^ cDC [25], while CD11b^+^CD11c^+^MHC-II^-^ cells resemble immature DC produced in splenic LTC and were termed L-DC [26]. From as early as 7 days, the cell yield in 5G3 co-cultures was noticeably higher than in 3B5 co-cultures. While the proportional distribution of subsets was similar for both stroma at 7 days, by 14 days 5G3 co-cultures produced 57% CD11b^+^CD11c^+^ myeloid DC compared with 3B5 co-cultures which produced only 9%. Also, 5G3 co-cultures produced a higher percentage of L-DC (CD11b^+^CD11c^+^MHC-II^-^) (24%), compared with 3B5 co-cultures (4%). This showed 5G3 to be the best supporter, and 3B5, a weak or non-supporter of myelopoiesis. These co-cultures produced few CD11b^+^CD11c^+^CD45RB^+^ cells, described by others as regulatory DC [27].

### Identification of specific and upregulated genes in 5G3

Transcriptome analysis was performed to identify genes specifically expressed or upregulated in 5G3 stroma compared with 3B5, to identify specific regulators of *in vitro* hematopoiesis. Affymetrix Murine Genome 430v2.0 genechips were employed and duplicate experiments conducted. Several hypotheses were investigated. Firstly that supporting stroma like 5G3 express genes which encode molecules important for cell-contact dependent signalling leading to hematopoietic differentiation. The second was that 5G3 secretes cytokines or chemokines which specifically support hematopoiesis. Comparative analysis across two experiments involved normalisation of genechips and removal of variation due to batch effect.

**Fig 2:**
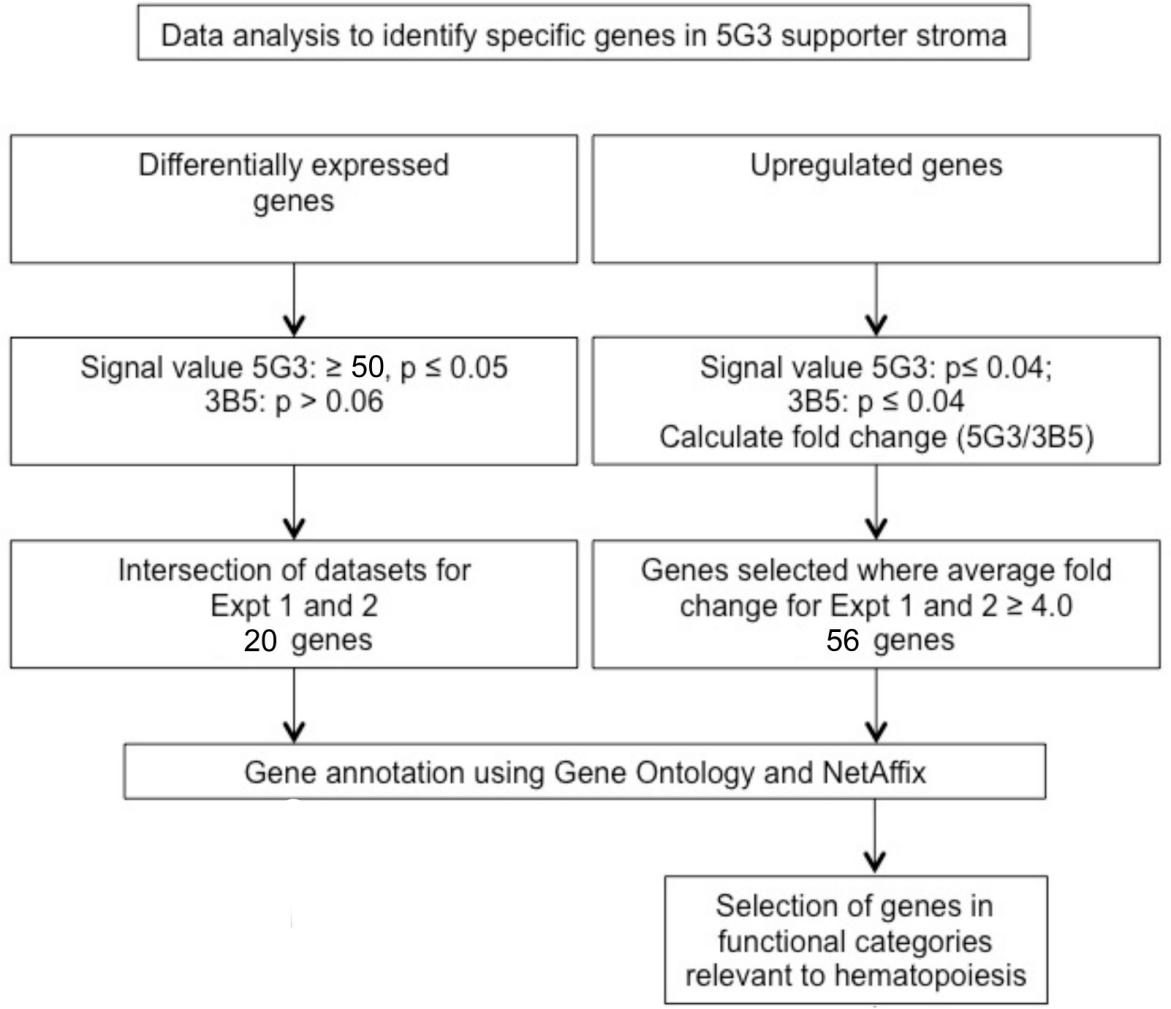
Procedure for identification of gene expression in stromal lines. Transcriptome analysis of 5G3 and 3B5 stroma employed Affymetrix Murine Genome 430v2 genechips. Two separate experiments were performed and data normalised for comparison of replicate samples. MAS5.0 (Affymetrix) software was used to standardize genechips and to calculate a signal value and p-value statistic for all probe sets. Gene annotation and literature searches were performed to identify genes of interest and to make predictions about gene function.

**Fig 3:**
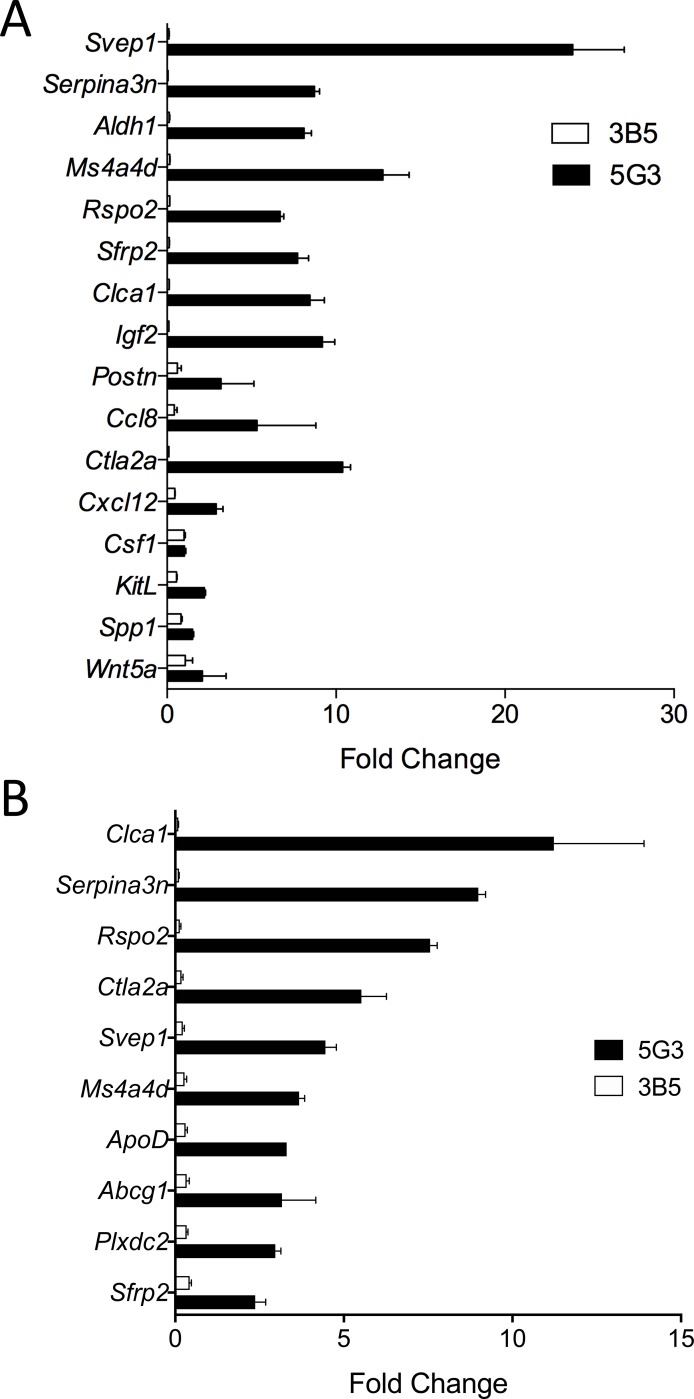
Quantitation of gene expression differences. Realtime PCR was performed across two separate experiments (A and B) to measure expression of selected genes determined by transcriptome analysis as upregulated or specifically expressed in 5G3 over 3B5. Also in (A), several genes related to hematopoiesis were shown to be expressed by both 5G3 and 3B5. These included *Cxcl12*, *Csf1*, *KitL*, *Spp1* and *Wnt5a*. Gene expression was quantified in terms of fold change (mean ± SE; n = 2) relative to of *Actb* (β-actin), a control housekeeping gene. Expression of genes by 5G3 was significantly different to 3B5 (p≥0.05) for all genes except *Csf1 and Wnt5a*.

Specific criteria were established to identify differentially expressed genes (Fig 2). A p-value of ≤0.04 was set to detect genes expressed by 5G3, and a p-value of >0.06 to detect genes not expressed by 3B5. The signal value threshold was set at ≥50. Datasets were obtained for each of two experiments and selection of genes across both experiments gave a dataset of 20 genes. Table 3 lists these genes and their signal values for 5G3 expression. *Apod*, *Svep1*, *Plxdc2*, *Aldh1*, *Serpina3n* and *Abcg1* were selected, on the basis of high signal value and potential role in hematopoiesis, for verification of differential gene expression using Realtime PCR (Fig 3). This confirmed that these 6 genes were expressed in significantly higher levels in 5G3 above 3B5.

**Table 3:** Identification of genes specifically expressed in 5G3 but not 3B5.

Probe set	Gene Symbol	Gene Title	Signal value*	
			Expt 1	Expt 2
1416371_at	*Apod*	apolipoprotein D	1694.3	1327.6
1419182_at	*Svep1*	sushi, von Willebrand factor type A, EGF and pentraxin domain containing 1	1206.7	758.3
1419100_at	*Serpina3n*	serine (or cysteine) peptidase inhibitor, clade A, member 3N	821.9	821.9
1451440_at	*Chodl*	chondrolectin	577.2	116.1
1418912_at	*Plxdc2*	plexin domain containing 2	298.3	280.0
1426758_s_at	Meg3**	maternally expressed 3	283.8	243.3
1416468_at	*Aldh1a1*	aldehyde dehydrogenase family 1, subfamily A1	277.8	591.1
1435261_at	*Tmtc1*	transmembrane and tetratricopeptide repeat containing 1	206.8	156.0
1418672_at	*Akr1c13*	aldo-keto reductase family 1, member C13	177.3	218.0
1417577_at	*Trpc3*	transient receptor potential cation channel, subfamily C, member 3	168.4	169.6
1424032_at	*Hvcn1*	hydrogen voltage-gated channel 1	166.1	282.6
1418603_at	Avpr1a**	arginine vasopressin receptor 1A	161.4	235.1
1450243_a_at	*Rcan2*	regulator of calcineurin 2	128.0	135.4
1423570_at	*Abcg1*	ATP-binding cassette, sub-family G (WHITE), member 1	111.5	96.0
1423266_at	*Fam123b*	family with sequence similarity 123, member B	93.0	88.0
1453317_a_at	*Khdrbs3*	KH domain containing, RNA binding, signal transduction associated 3	80.6	132.8
1436736_x_at	Nrep**	neuronal regeneration-related protein	79.3	78.4
1451716_at	*Mafb*	v-maf musculoaponeurotic fibrosarcoma oncogene family, protein B (avian)	73.2	95.1
1450492_at	*Cngb3*	cyclic nucleotide gated channel beta 3	70.2	52.4
1429444_at	*Rasl11a*	RAS-like, family 11, member A	48.9	114.5

*Gene expression reflects signal value (MAS5.0 calculation) in 5G3 (p ≤ 0.04, signal value ≥ 50) for genes where signal is absent for 3B5

(p > 0.06). Two independent experiments were performed. Genes listed in order of expression level for Expt 1.

**For these genes, two probe sets returned similar results, but only one representative probeset is shown.

**Fig 4:**
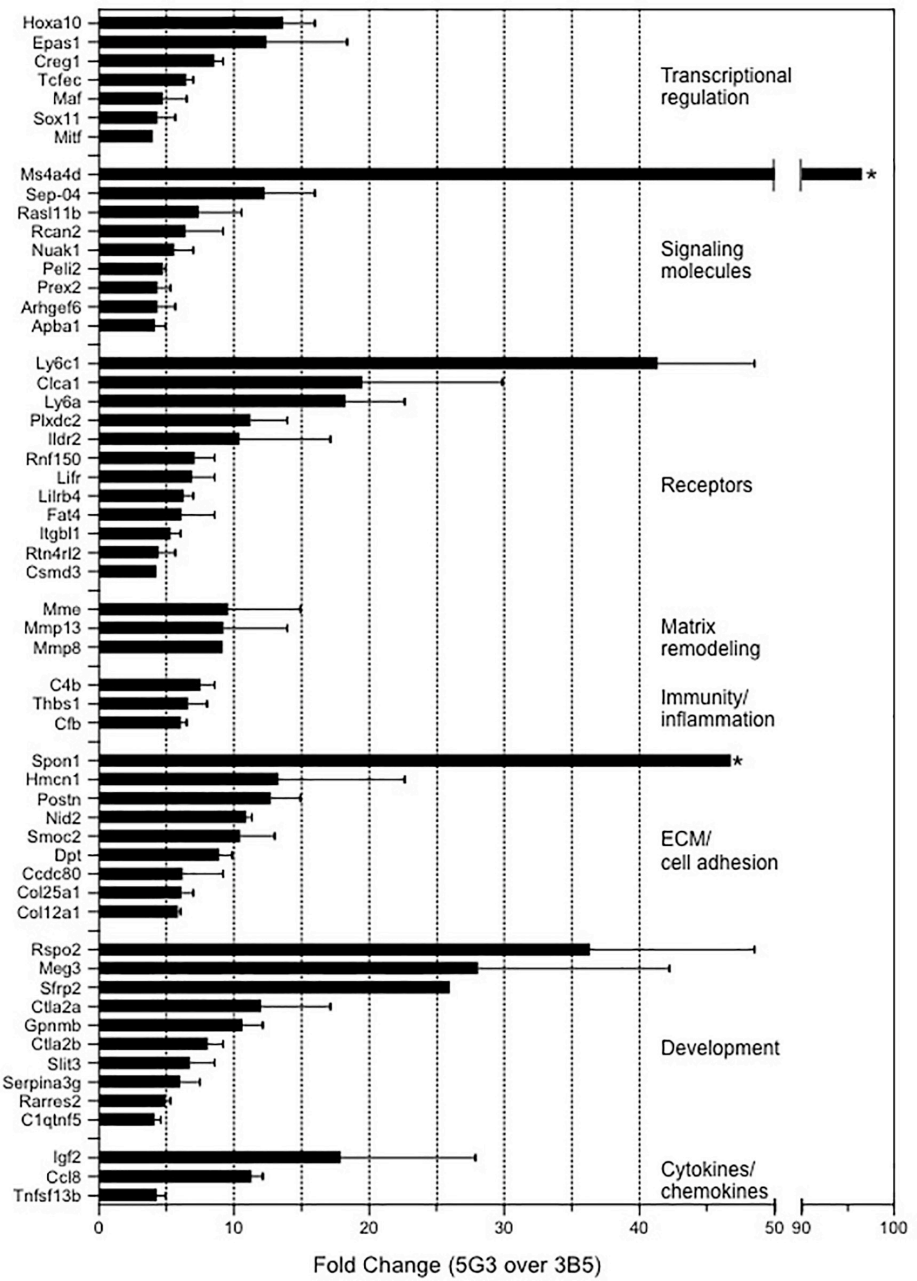
Genes upregulated in 5G3 over 3B5. Data represent average fold change in gene expression for 5G3 over 3B5 shown as mean ± S.E (n = 2). For the two most highly upregulated genes identified by *, SE is not shown but reflects 30–40% of the mean. Genes were annotated using the Gene Ontology ‘molecular function’ tool and only genes in categories associated with the role of stroma in hematopoiesis were selected.

Criteria imposed to select genes upregulated in 5G3 over 3B5 are described in Fig 2. This analysis selected genes present in both 5G3 and 3B5 as defined by the detection call in the MAS5.0 software. Fold change was calculated as the ratio of 5G3 over 3B5 signal values. Eighty-eight genes with an average fold change of ≥4.0 were identified. Genes were then grouped into functional categories using Gene Ontology, and functional categories relevant to hematopoiesis were identified. Categories selected included transcriptional regulation, signalling molecules, receptors, matrix remodelling, immunity and inflammation, extracellular matrix/cell adhesion, development and cytokines/chemokines. This gave 56 genes of interest (Fig 4). Several genes with a potential role in hematopoiesis were then selected for further quantification using Realtime PCR. These included: *Ms4a4d*, *Clca1*, *Plxdc2*, *Postn*, *Rspo2*, *Sfrp2*, *Ctla2α*, *Igf2 and Ccl8* (Fig 3).

Affymetrix profiling indicated the common expression by both 5G3 and 3B5 of several genes which encode known regulators of hematopoiesis, including *Cxcl12*, *Csf1*, *KitL*, *Spp1*, *Vcam1* and *Wnt5a*, and Realtime PCR was used to confirm gene expression (Fig 3). Since these genes were all commonly expressed by 5G3 and 3B5 stroma, they are not considered to be deterministic of hematopoiesis in this *in vitro* system. However, expression of *Cxcl12*, *KitL and Spp1*, but not *Csf1* or *Wnt5a*, was significantly higher in 5G3 than in 3B5.

### Growth factors as regulators of *in vitro* hematopoiesis

Genes of interest either upregulated or specifically expressed by 5G3 over 3B5 included soluble factors available as commercially recombinant molecules in highly purified form (Table 1). The possibility was tested that addition of these to 3B5 co-cultures of Lin^-^ bone marrow cells over 3B5 may restore hematopoietic support capacity. This experiment was conducted bearing in mind the possibility that one factor alone may not be sufficient to restore hematopoietic support function to 3B5. Lin^-^ bone marrow progenitors were overlaid on 3B5 stroma, and co-cultures maintained for 14 days. Every 4 days, co-cultures were supplemented with recombinant factors at concentrations of 0, 0.03, 0.1 and 0.3 μg/mL. Cell counting and flow cytometric analysis was used to quantitate cells produced in co-cultures. As in Fig 1, myeloid cells are identified as CD11b^+^CD11c^-^ and DC as CD11b^+^CD11c^+^ cells. Progenitors cells would be present within the CD11b^-^CD11c^-^ fraction. In these experiments it was very difficult to isolate sufficient cell numbers to do many replicates within the one experiment. Therefore, a titration of additives was used, such that a significant inhibitory (or enhancing) effect was determined by the outcome of decreasing cell (or increasing) production over 4 dilutions of added factor.

**Fig 5:**
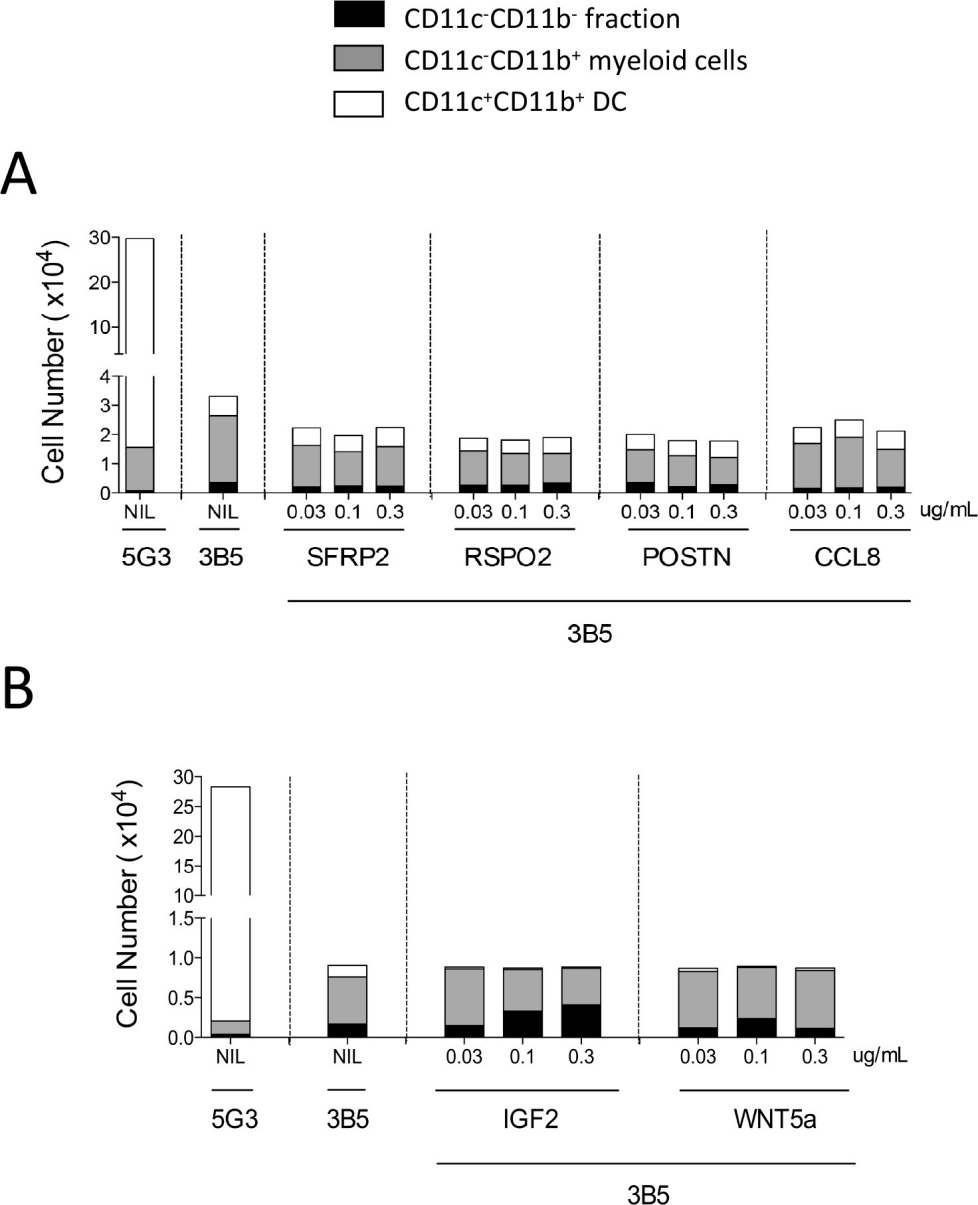
Addition of growth factors to co-cultures established over 3B5. Lin^-^ bone marrow was overlaid on 3B5 stroma and cultured in the presence and absence of recombinant factors. SFRP2, RSPO2, IGF2, WNT5a, CXCL12 and CCL8 (Table 1) were added to cultures at 0, 0.03, 0.1 and 0.3 μg/mL. Every 4 days, a half volume of culture medium was replaced with complete medium containing factors. Two separate experiments were performed (A and B). Non-adherent cells were collected on Day 14 and stained with fluorochrome-conjugated antibodies specific for CD11b, CD11c, MHC-II, and F4/80. Propidium iodide (PI: 1 μg/mL) staining was used to gate PI^-^ live cells. ‘Fluorescence minus one’ controls were used to set gates to identify specific antibody binding. Myeloid cells were gated as CD11b^+^CD11c^-^ cells, DC as CD11b^+^CD11c^+^ cells, and CD11b^-^CD11c^-^ cells were gated as the fraction enriched for progenitors. MHC-II expression was used to confirm identify of subsets (not shown). Data are presented as total number of each cell type produced in co-cultures. No statistically significant enhancement of growth was identified across a 4-point titration assay for any growth factor tested.

While 5G3 co-cultures produce a high yield of cells with a predominance of DC, 3B5 produce at least 10-fold fewer cells with a predominance of myeloid cells (Fig 5A and 5B). Addition of SFRP2, RSPO2, POSTN and CCL8a had minimal effect on cell production and no noticeable effect on the relative proportion of cells produced in 3B5 co-cultures. None of these factors alone was able to restore the high cell production typical of 5G3. While IGF2 and WNT5a are known regulators of hematopoiesis, addition to 3B5 failed to increase cell yield in co-cultures. However, IGF2 addition produced a significant concentration-dependent increase in the CD11b^-^CD11c^-^ fraction containing progenitors with a reduction in myeloid cell production (Fig 5B). This suggests a distinct role for IGF2 in the proliferation of progenitors, consistent with previous reports for its role in stem cell proliferation [28–30]. While IGF2 addition failed to restore cell production to 5G3 levels, it did impact the hematopoietic events underway in 3B5. Several experiments also involved addition of all factors in combination used at the same concentration as individually. This gave no change in the production of cells compared with unsupplemented co-cultures.

### Specific regulators of hematopoiesis

Based on specific and upregulated gene expression by 5G3, several available reagents were tested as inhibitors of hematopoiesis supported by 5G3 stroma. Some of these reagents have unique binding specificity for adhesion molecules and growth factors specifically expressed by 5G3 but not 3B5. Others are less specific in their binding to the molecules of interest. The inhibitors tested and their target molecules are listed in Table 2. Co-cultures were established using Lin^-^ bone marrow overlaid on 5G3 stroma. With the difficulty of isolating progenitors, we chose to assess inhibition of cell production across a range of inhibitor concentrations such that significant effects could be established as a dilution across a 4 point titration. All inhibitors were specific for cell surface molecules. However, CXCR7 was shown to be expressed mainly as an intracellular molecule, although its function could be inhibited by addition of antibodies to culture medium [31]. Inhibitors added to co-cultures were replenished every 4 days at medium change. Two independent experiments were conducted to test 7 different inhibitors.

**Fig 6:**
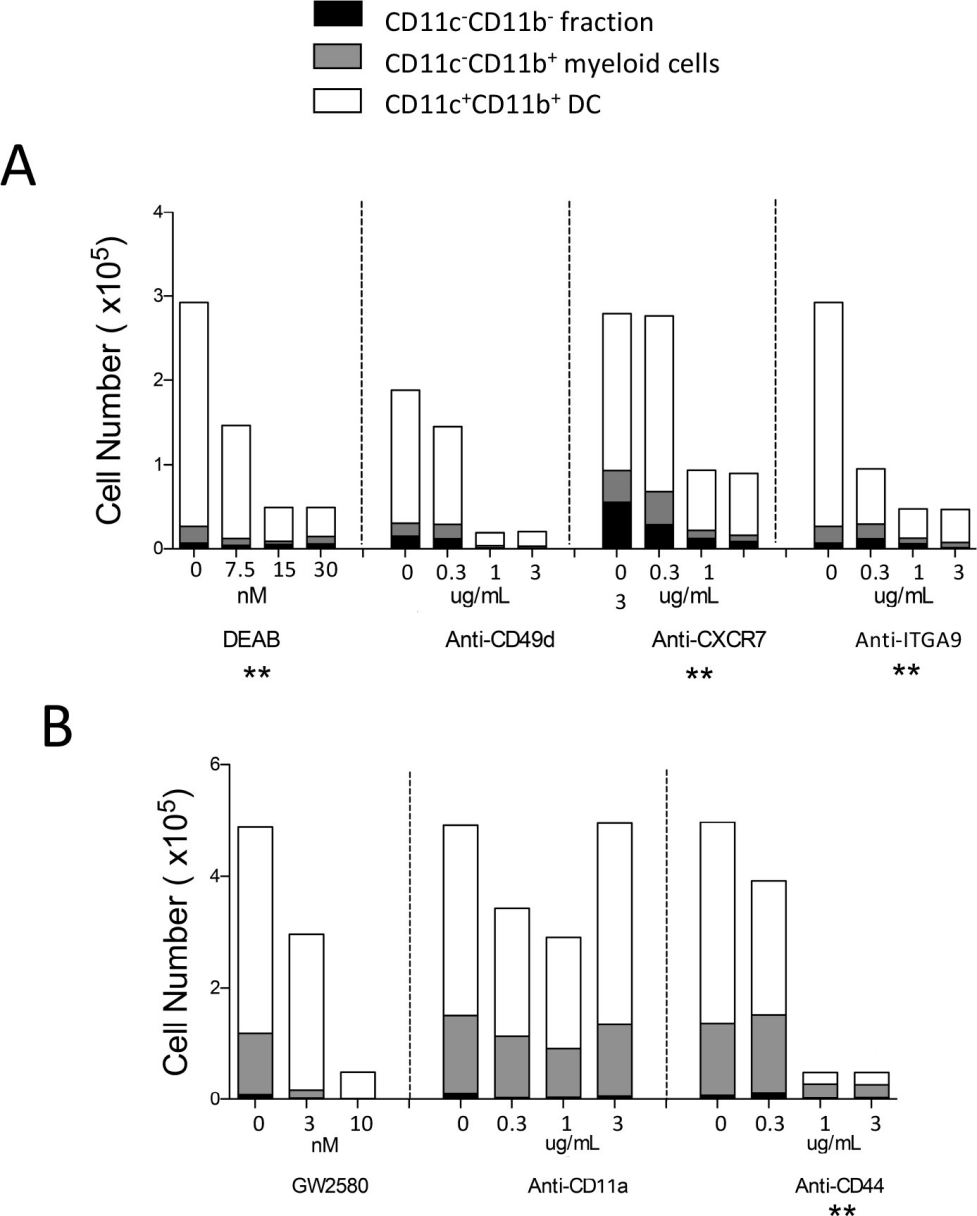
Assessment of potential inhibitors of hematopoiesis. Co-cultures were established by overlay of 2.10^4^ Lin^-^ bone marrow cells on 5G3 stroma in the presence and absence of inhibitors. Every 4 days, a half volume of medium was replaced with medium containing inhibitor. Two experiments (A and B) were performed. Inhibitors included DEAB, GW2580, and antibodies to CXCR7, CD11a, CD44, SPP1 and CD49d as listed in Table 2. Control (Con) co-cultures contained no inhibitor or an isotype control antibody used at the highest antibody concentration for antibody. Non-adherent cells were collected on Day 14 and stained with fluorochrome-conjugated antibodies specific for CD11b, CD11c, MHC-II and F4/80. Propidium iodide (PI: 1 μg/mL) staining was used to gate PI^-^ live cells. ‘Fluorescence minus one’ controls were used to set gates to identify specific antibody binding. Myeloid cells were gated as CD11b^+^CD11c^-^ cells, DC as CD11b^+^CD11c^+^ cells, and CD11b^-^CD11c^-^ cells were gated as the fraction enriched for progenitors. MHC-II expression was used to confirm identify of these subsets (not shown). Data are presented as total number of each cell type produced in co-cultures. Statistically significant inhibition (p≥0.05) was identified through a consistent dilution effect across a 4-point titration and is shown as **.

DEAB is a specific inhibitor of aldehyde dehydrogenase including both ALDH1A1 and ALDH2 [32], although only ALDH1A1 was found to be specifically expressed by 5G3 with both 5G3 and 3B5 expressing ALDH2 (S1 Table). However, when used here as an inhibitor of 5G3 co-cultures, Diethylaminobezaldehyde (DEAB) produced a significant concentration-dependent reduction in cell production, coincident with reduction in number of myeloid and dendritic cells produced (Fig 6A). In order to determine whether SVEP1, expressed uniquely by 5G3 and not 3B5, is deterministic of hematopoiesis in co-cultures, antibody specific for ITGA9 was added into co-cultures. Integrin α_9_β_1_ expressed by HSC is a known binding receptor for SVEP1 [33, 34]. A significant concentration-dependent reduction in cell production was detected (Fig 6A), suggesting that this interaction is critical despite the fact that Integrin α_9_β_1_ also binds to other molecules including FN, SPP1, ADAMs and TNC [35]. Affymetrix data mining has shown that transcripts for both of these molecules are expressed by 5G3 and 3B5 (S1 Table). CLCA1 is uniquely expressed by 5G3 and not 3B5. Inhibition of function tests in involved antibody to the CLCA1 ligand CD11a [36]. However CD11a also binds to several other molecules including ICAMs and JAM-A [37, 38], although these were not found to be expressed by 5G3 or 3B5 stroma (S1 Table). Since this antibody had little effect on cell production in co-cultures, CLCA1 would not appear to be a candidate regulator of hematopoiesis (Fig 6B).

Further inhibition assays involved genes of interest due to their previously described roles in regulating hematopoiesis. These included *Cxcl12*, *Vcam1*, *Csf1 and Spp1* and were all shown to be expressed by both 5G3 and 3B5 (S1 Table). CXCL12 is produced by stroma is known to bind to both CXCR4 and CXCR7 expressed by HSC [39]. Evidence that antibody to CXCR7 inhibited cell production in co-cultures supports a potential role for CXCL12 in *in vitro* hematopoiesis (Fig 6A). Since CD49d is a ligand for VCAM1 expressed by 5G3, antibody to CD49d was tested as an inhibitor (Fig 6A). Strong inhibition would suggest an important role for this adhesive interaction in hematopoiesis *in vitro*. A role for CSF1 was tested through addition of the GW2580 inhibitor of CSF1R to co-cultures. This reduced cell production in a concentration-dependent manner (Fig 6B), suggesting a requirement for CSF1. While a three point inhibition test was not significant, this result is supported by evidence for a role for CSF1 in myelopoiesis in spleen co-cultures identified previously [40]. SPP1(osteopontin) is a known regulator of hematopoiesis in bone marrow which binds to CD44 [41]. When antibodies to CD44 were added into co-cultures, a four point dilution in cell production indicated significant inhibition (Fig 6B). CD44 however also binds to several other molecules including hyaluronic acid and selectins, but there was no evidence from gene profiling that these genes were expressed by 5G3 stroma, and so were unlikely targets for antibody inhibition.

## Discussion

The selection of differentially expressed genes involved stringent criteria for expression in 5G3 and not 3B5. The aim was to identify genes with high certainty of differential expression. The selected genes in Table 3 represent a range of biological functions. These dictate functions of transport (*Abcg1*, *Apod*, *Cngb3* and *Hvcn1*), metabolism (*Akr1c13*), signalling (*Avpr1a*, *Rcan2* and *Rasl11a*), cell adhesion (*Svep1*), and development (*Serpina3n*, *Plxdc2*, *Meg3*, *Aldh1a1* and *Nrep*). Genes which dictate development or cell-cell contact are of primary interest, particularly those expressed in highest levels like *Apod*, *Svep1*, *and Serpina3n* and the proteins encoded by *Plxdc2* and *Aldh1a1*. The dominant expression of many of these genes in 5G3 over 3B5 was confirmed by Realtime PCR (Fig 3). The function of ALDH1a1 and SVEP1 in *in vitro* hematopoiesis is predicted through studies showing that specific inhibitors DEAB (for ALDH1a1) and anti-ITGA9 (for SVEP1) inhibited cell production in 5G3 co-cultures (Fig 6).

Precedence also exists in the literature for the involvement of SVEP1, SERPINa3n, and ALDH1a1 in HSC differentiation *in vivo*. Evidence for their involvement in the production of L-DC in stromal co-cultures is also consistent with previous evidence that HSC are direct progenitors of L-DC *in vitro* [11, 20, 42]. SVEP1 is of interest because of its novelty, and its involvement in cell adhesion [43]. Only recently it was shown that SVEP1 binds to ITGA9_,_ a member of the integrin family of molecules which regulate cell adhesion, migration and signalling [44]. A study by Schreiber *et al*. [34] showed that anti-ITGA9 antibodies absorbed to HSPC led to a 60% reduction in their adhesion to osteoblasts. Antibodies to integrin α_9_ also inhibited cell proliferation and differentiation in cytokine-supplemented cultures of HSPC. Addition of anti-ITGA9 antibody to 5G3 co-cultures studied here gave an overall reduction in cell production. SVEP1 may contribute to the adhesion of HSPC to stroma which is essential for myelopoiesis. SERPINa3n and SERPINa3g were found to be specifically expressed or upregulated by 5G3 stroma. SERPINa3 has previously been identified as an important regulator of HSC maintenance [45]. There are 14 replicates of SERPINa3 (a-n) in the mouse chromosome which function as protease inhibitors that control serine peptidases through cleavage and inactivation of molecules which retain HSC in the microenvironment, so influencing HSC mobilization [45]. ALDH1a1 is an enzyme that drives retinoic acid synthesis [46], and the retinoic acid signalling pathway has been shown to drive both LT-HSC and ST-HSC proliferation [47]. The importance of ALDH1a1 expressed by 5G3 stroma in HSC proliferation is indicated through inhibition with DEAB leading to a reduction in cell production.

The selection of upregulated genes involved criteria that genes be expressed in both 5G3 and 3B5, with an average fold change across two experiments of ≥4-fold. This protocol selected genes within functional categories related to the hematopoietic support capacity of stroma. Genes most highly upregulated, included *Hoxa10*, *Ms4a4d*, *Clca1*, *Plxdc2*, *Spon1*, *Postn*, *Rspo2*, *Sfrp2*, *Ctla2*, *Igf2 and Ccl8*. These genes should provide the molecular interactions between stroma and HSC necessary for maintenance of HSC in the quiescent state, HSC self-renewal, and cell fate decisions leading to proliferation, signalling for stem cell migration, and further differentiation.

Adhesion molecules are of particular interest in terms of regulation of hematopoiesis in stromal co-cultures. CLCA1 binds to LFA-1, an adhesion molecule expressed on HSC [48]. While CLCA1 is highly upregulated in 5G3 stroma, and is also expressed on lymphatic endothelial cells in spleen [49], we were unable to demonstrate a functional role in hematopoiesis *in vitro*. In terms of regulators of early hematopoiesis, secreted proteins RSPO2 and SFRP2 are known regulators of Wnt signalling, particularly involving WNT3a, which is central to HSC self-renewal [50]. They bind to WNT3a with opposite outcomes. RSPO2 amplifies WNT3a signalling, promoting HSC maintenance and self-renewal [51], while SFRP2 negatively regulates WNT3a [52, 53], so stimulating HSC proliferation [54]. Dynamic interaction between these two proteins may be key to switching HSC between self-renewal and proliferation. These molecules may play an important role in *in vitro* hematopoiesis since they are highly upregulated in 5G3 over 3B5 stroma. However, addition to 3B5 co-cultures was insufficient to trigger hematopoiesis, possibly since important adhesion molecules were also not expressed by 3B5. Another soluble factor, chemokine CCL8, was found to be upregulated in 5G3 over 3B5. CCL8 cooperation with CXCL12 is known to attract hematopoietic progenitors with a potential to differentiate into regulatory DC [55]. The role of CXCL12 in HSC maintenance is well documented [56], and both 5G3 and 3B5 express high levels. Hence, interaction between these two chemokines, in the context of hematopoiesis supported by 5G3 stroma, indicates potential for production of regulatory DC in stromal co-cultures. Recent work from this lab has identified transient production of regulatory DC in co-cultures, but clearly distinguishes them from L-DC which are continuously produced [57].

This report also establishes the expression and importance of genes which are known regulators of hematopoiesis and the production of myeloid cells in 5G3 stromal co-cultures. CSF1 is a known regulator of myelopoiesis from *in vivo* studies in mice. The injection of CSF1 into C57BL/6 mice promotes DC development from common myeloid progenitors with a resultant 2-fold increase in splenic pDC and cDC numbers [58]. Previous studies in this lab revealed an important role for CSF1 in the development of cDC-like cells, but not L-DC, in co-cultures of Lin^-^ bone marrow over 5G3 stroma [20, 40]. The CSF1R inhibitor, GW2850, had a strong inhibitory effect on the production of myeloid cells. It is possible therefore that CSF1 produced by 5G3 acts as a growth factor to promote myeloid cell development, and cDC-like cell production in Lin^-^ bone marrow co-cultures.

Antibody to CD44 was also a very effective inhibitor of cell production in co-cultures. The target of inhibition appears to be specifically L-DC. This suggests that SPP1 (osteopontin), a ligand for CD44 expressed by 5G3, may play a fundamental role in L-DC development. SPP1 is known to maintain HSC in the quiescent state by promoting engraftment to the stem cell niche [59, 60]. SPP1 expressed by 5G3 may act to regulate and maintain progenitors in co-cultures, such that when SPP1 function is inhibited with antibody, more progenitors differentiate, and their numbers become exhausted. This would lead to a reduction in the differentiation of cells, and particularly L-DC production which are known to arise by direct differentiation from HSC and MPP [20, 40].

Several known regulators of *in vivo* hematopoiesis have been identified in this study. CXCL12 binds to CXCR4 and CXCR7 which are receptors on HSPC [41]. While CXCR7 was not shown to be expressed as a cell surface receptor on HSC in this study, it must be expressed transiently, since anti-CXCR7 antibodies gave a noticeable reduction in cell production. The importance of VCAM1 expressed by 5G3 was demonstrated through anti-CD49d inhibition of cell production in co-cultures. The CD49d-VCAM1 interaction promotes cell adhesion, and so VCAM1 on 5G3 may be important for adhesion of progenitors to stroma where it acts as a regulator of proliferation and differentiation. This finding is supported by evidence that stem cell mobilising agents applied to *Vcam1*^-/-^ mutant mice gave an increase in circulating CFU-C over wild type mice, with a decrease in CFU-C accumulation in bone marrow and spleen [61].

Several other molecules identified reflect potential regulators of *in vitro* hematopoiesis. CTLA2 is expressed highly in the trabecular bone region of the bone marrow, suggesting a potential role in hematopoiesis [62]. PLXDC2, is unknown in its role in hematopoiesis, but is well-documented as a mitogen that drives neural stem cell proliferation [63]. These molecules together with MS4A4D are relatively unexplored in the context of hematopoiesis, but their upregulation in 5G3 over 3B5 makes them interesting targets for further study.

## Conclusion

The cloned stromal cell line 5G3 is a supporter of hematopoiesis while 3B5 is a non-supporter. 5G3 supports production of dendritic-like cells as well as myeloid cells and progenitors, while 3B5 transiently supports the production of only low numbers of myeloid cells. Gene expression profiling was performed using Affymetrix genechips to compare gene expression between 5G3 and 3B5 stroma and to identify genes which regulate this process. Initially, data mining identified gene expression essential for hematopoiesis in bone marrow including *Cxcl12*, *KitL*, *Spp1*, *Vcam1* and *Csf1*. Hematopoiesis is responsible for the production of myeloid cells, and this is supported by evidence that antibodies or inhibitors of several of these gene products which act through blocking receptors (anti-CXCR7 for CXCL12; anti-CD49 for VCAM1; and GW2580 for CSF1R) led to reduced cell production of in 5G3 co-cultures. Such evidence justifies the search for differentially expressed or upregulated genes in 5G3 over 3B5 as determinants of *in vitro* hematopoiesis, and verifies the model co-culture system as highly suitable for returning genes which determine the hematopoietic support environment of HSC.

## Supporting information

S1 Table Gene expression in 5G3 stroma. (DOCX)
