# Supplementary material for: Identification of genes which regulate stroma-dependent in vitro hematopoiesis
Source: PLoS One. 2018 Oct 11;13(10):e0205583. doi: 10.1371/journal.pone.0205583 (PMC6181386; doi:10.1371/journal.pone.0205583)
Supplement: S1 Table — (DOCX) [file pone.0205583.s001.docx]

| **Probe set** | **Gene symbol** | **Gene title** | **Signal value*** | | | |
| --- | --- | --- | --- | --- | --- | --- |
|  |  |  | **5G3-1** | **5G3-2** | **3B5-1** | **3B5-2** |
| **1416468_at** | ***Aldh1a1*** | **aldehyde dehydrogenase family 1, subfamily A1** | **534.7** | **1555.2** | **50.7** | **33.3** |
| 1448143_at | *Aldh2* | Aldehyde dehydrogenase 2 | 1466.4 | 1692.1 | 1390.1 | 1079.4 |
| 1448162_at | *Vcam1* | vascular cell adhesion molecule 1 | 4074.2 | 4531.2 | 3968.0 | 4674.9 |
| 1426642_at | *Fn1* | fibronectin 1 | 7215.6 | 4360.3 | 9341.9 | 5830.5 |
| 1417574_at | *Cxcl12* | chemokine (C-X-C motif) ligand 12 | 3690.2 | 2770.8 | 2522.8 | 1625.5 |
| **1419182_at** | ***Svep1*** | **sushi, von Willebrand factor type A, EGF and pentraxin domain containing 1** | **2316.8** | **1347.1** | **84.1** | **69.1** |
| 1449254_at | *Spp1* | secreted phosphoprotein 1 | 10038.1 | 9399.7 | 11250.5 | 10663.6 |
| 1416342_at | *Tnc* | tenascin C | 3896.3 | 4149.4 | 4259.6 | 3171.8 |
| 1443377_at | *Adam1a* | a disintegrin and metallopeptidase domain 1a | 38.9 | 40.3 | 38.9 | 40.3 |
| 1460374_at | *Adam2* | a disintegrin and metallopeptidase domain 2 | 16.3 | 18.4 | 20.4 | 16.0 |
| 1448329_at | *Adam3* | a disintegrin and metallopeptidase domain 3 (cyritestin) | 18.7 | 21.3 | 18.7 | 21.3 |
| 1460220_a_at | *Csf1* | colony stimulating factor 1 (macrophage) | 1681.8 | 1973.7 | 2965.4 | 3939.5 |
| **1417852_x_at** | ***Clca1*** | **chloride channel calcium activated 1** | **2520.0** | **1923.6** | **237.1** | **102.3** |
| 1424067_at | *Icam1* | intercellular adhesion molecule 1 | 18.4 | 17.5 | 17.5 | 21.0 |
| 1448862_at | *Icam2* | intercellular adhesion molecule 2 | 16.4 | 15.8 | 22.3 | 19.4 |
| 1424595_at | *F11r* | F11 receptor (JAM-A) | 65.4 | 79.0 | 39.5 | 38.4 |
| 1449169_at | *Has2* | hyaluronan synthase 2 | 62.7 | 46.8 | 255.4 | 119.3 |
| 1421712_at | *Sele* | selectin, endothelial cell | 18.0 | 21.5 | 29.7 | 39.3 |
| 1455940_x_at | *Sell* | selectin, lymphocyte | 15.8 | 19.8 | 20.7 | 17.8 |

**S1 Table. Gene Expression in 5G3**

*Data mining of Affymetrix datasets was used to compile signal values (MAS5.0 calculation) for genes of interest reflecting gene expression in duplicate samples of 5G3 stroma. Genes expressed specifically or upregulated (>3fold) in 5G3 over 3B5 are shown in red.
